# Malaria knowledge and utilization of chemoprophylaxis in the UK population and in UK passengers departing to malaria-endemic areas

**DOI:** 10.1186/1475-2875-12-461

**Published:** 2013-12-21

**Authors:** Ron H Behrens, Neal Alexander

**Affiliations:** 1 Department of Clinical Research, Faculty of Infectious and Tropical Diseases, London School of Hygiene and Tropical Medicine, London, UK; 2 Hospital for Tropical Diseases, UCLH NHS Foundation Trust, London, UK; 3 MRC Tropical Epidemiology Group, Department of Infectious Disease Epidemiology, Faculty of Epidemiology and Population Health, London School of Hygiene and Tropical Medicine, London, UK

**Keywords:** Travellers, Malaria, Chemoprophylaxis, Risk perception, Compliance, VFRs

## Abstract

**Background:**

The burden of imported malaria is predominantly in travellers visiting friends and relatives (VFR) in sub-Saharan Africa. The failure of this group to use chemoprophylaxis is recognized as the most important risk factor for the high incidence of disease. Understanding the reasons for failure to follow national recommendations may relate to knowledge, risk perception, cost, and peer pressure. Research into these variables is critical to understand and change practices in this group and this study was designed to explore whether knowledge, risk perception and prophylaxis use differs between travellers’ to various destinations and the rest of the UK population.

**Methods:**

Two face-to-face questionnaire surveys were conducted to collect information on demographics, malaria knowledge, source, and quality of pre-travel advice, past travel experience and perceived malaria threat. One was an IPSOS survey of individuals representative of the UK population. The other was a departure lounge survey (Civil Aviation Authority (CAA)) of passengers departing to malarious regions detailing destinations and use of chemoprophylaxis.

**Results:**

Around a quarter of the 1,991 UK population surveyed had previously travelled to a malarious area. Five-hundred departing passengers were interviewed, of which 80% travelled for leisure (56% VFR’s) and 42% were travelling to West Africa. Malaria knowledge among the UK population (score 58.6) was significantly lower than that of individuals who had previously travelled or were travelling (63.8 and 70.7 respectively). Malaria knowledge was similar in individuals who had and had not sought pre-travel advice and travellers using and not using chemoprophylaxis for their journey. Leisure travellers to Ghana and Nigeria were predominantly VFRs (74%), whilst 66% of travellers to Kenya were tourists. Despite similar high knowledge scores and perceived (>90%) threat of the lethality of malaria in the three groups, chemoprophylaxis use in Nigerians (50%) was substantialy lower than in passengers departing to Kenya (78%) and Ghana (82%). More frequent annual return visits were made to Nigeria (72%) than to Ghana (38%) or Kenya (23%).

**Conclusion:**

Travellers had more malaria knowledge than the non-travelled UK population. Malaria knowledge, perceived threat, travel experience, and quality of pre-travel advice appear unrelated to the use of chemoprophylaxis in passengers. Reducing malaria in VFR travellers will require strategies other than improving malaria knowledge and enhancing malaria risk awareness.

## Background

Malaria is an important infectious disease: the World Health Organization (WHO) estimates 219 million cases of malaria (range 154–289 million) and 660,000 deaths (range 610,000-971,000) in 2010 [1]. Worldwide travel, exposing travellers from non-endemic regions to malaria-endemic countries, is an ongoing source of imported malaria. In the UK approximately 1,500 cases are imported annually of which 76% are *Plasmodium falciparum* malaria and the majority (64.5%) of imported cases are acquired by travellers visiting friends and relatives (VFRs), and around half (54%) is acquired during travel to either Ghana or Nigeria of whom only 7% use malaria preventative measures [2].

Understanding why the currently very effective, available measures are not used by high-risk travellers is fundamental to reducing the burden of imported disease. The challenge of studying knowledge, attitudes and practices (KAP) of travellers is in capturing the information from an unbiased and representative population, and not relying on data from those seeking pre-travel health advice or questioning patients having developed malaria after travelling. Some studies have focussed on high-risk VFR travellers. Pistone and colleagues assessed the KAP of VFRs around malaria transmission but these were recruited in travel clinics [3], and Leonard and Van Landingham explored practices using discussion groups of Nigerians who had previously travelled to Nigeria and were recruited from a community in Houston, USA [4]. Schilthuis [5] interviewed West African immigrants, from countries including Nigeria and Ghana, living in the Netherlands who were recruited from a community through churches and local societies. Van Genderen and colleagues explored changing KAP in departing passengers from Schiphol Airport between 2002 and 2009 to understand educational needs for Dutch travellers on malaria [6].

This study was designed to investigate the knowledge and practices of a representative sample of the population by interviewing 2,007 of the UK population of which 1991 were completed. A similar tool was used to question passengers in departure lounges at Heathrow Airport whose destination was a malaria-endemic country. This would allow a comparison of the knowledge and practices of the two populations and understand what factors might influence knowledge, including variables such as previous travel experience and sources of pre-travel information and demographics (age, income and reason for travel). The departure lounge survey also allowed for interviewing an unbiased travelling population to malaria-risk regions from which to identify the proportion of travellers using chemoprophylaxis.

## Methods

### UK population sampling

Adults aged 15 years or more were interviewed at home using IPSOS Mori’s Capibus survey during a five-day period at the end of May 2011. This uses a two-stage method, the primary sampling units (PSU) being amalgamations of the 2001 UK Census Output Areas, each of which has approximately 125 addresses. The primary sampling units are randomly selected and within them randomly selected secondary sampling units are contiguous zones made up of Output Areas, usually two of them. This yields a random sample of 2,007 anonymous respondents, representative of the population at a national and regional level.

The standard tool was supplemented by a malaria-specific questionnaire covering knowledge of transmission, clinical features, severity and outcomes, and methods of prevention. Information on past travel and malaria destinations and willingness to pay for malaria prevention was also captured.

Airport departure lounge sampling was undertaken by trained interviewers through a face-to-face survey conducted by the Civil Aviation Authority (CAA). Five-hundred British nationals travelling to sub-Saharan Africa or other malaria-endemic countries, as defined in the UK’s Advisory committee on Malaria Prevention (ACMP) guidelines [7], were interviewed prior to boarding their flight between June and August 2011. A multistage sampling design was employed on those who were randomly selected for interview and completed an anonymous CAA passenger questionnaire [8] with added questions on malaria, similar to those used in the IPSOS survey, supplemented with additional information on chemoprophylaxis use for the journey, source(s) of pre-travel health advice and household income.

### Analysis

The data were cleaned and imported into R where descriptive tables were created. Analysis of the IPSOS data used the survey weights provided. For each of the two surveys, a malaria knowledge score was based on four questions relating to the mode of transmission, symptoms, severity, and curability of malaria. For the first two questions, multiple options were offered and negative points were assigned for incorrect answers. For the other two questions, a single option was chosen from a list, each option being assigned zero or a positive number of points. The score for each question was scaled to 25, resulting in a maximum knowledge score of 100.

Sources of pre-travel advice were categorized according to whether this was from a health source, non-health source (internet or friend) or no pre-travel advice. Within each survey, differences in knowledge score were calculated between subgroups. This was done using parametric methods (*t* test and regression) because initial comparisons with bootstrap gave very similar results, suggesting that they were sufficiently robust. Where 95% confidence intervals for a difference overlapped between the two surveys, a meta-analysis combined estimate was also calculated [9].

## Results

The IPSOS Mori survey interviewed 2,007 individuals on 28 and 29 May 2011, selected as a representative sample of the UK general population; 1,991 interviews were completed and included for further analysis. Of these, 548 had previously visited a malaria-endemic country and were analysed as a separate group. The CAA survey included 499 departing passengers to malaria-endemic countries, with some data missing on ethnicity and reason for travel.

The age, gender, socio-economic class and household income are presented in Table 1.

**Table 1:** Demographic and descriptive information

	**CAA**^**a**^		**IPSOS**^**b**^			
			**Complete survey group**		**Travelled to malaria-endemic area**	
Gender, n male:female (%)	264:235	(53:47)	996:995	(48:52)	291:257	(52:48)
Age in years: mean (SD)^c^ 15-24	126	(25)	311	(16)	56	(9)
25-34	96	(19)	294	(16)	92	(18)
35-44	88	(18)	312	(18)	109	(24)
45-54	92	(19)	305	(16)	90	(17)
55-64	65	(13)	322	(14)	90	(15)
≥65	27	(5)	434	(20)	109	(18)
Socioeconomic group: n (%)^d^ A	31	(7)	69	(4)	38	(8)
B	121	(27)	383	(23)	158	(34)
C1	215	(47)	696	(29)	200	(29)
C2	53	(12)	387	(21)	81	(16)
D	28	(6)	266	(15)	47	(9)
E	6	(1)	190	(8)	24	(3)
Annual income (£, median class)^e^	28,750-34,499		25,000-29,999		30,000-39,999	
*Total*	499		1991		548	

^a^1 person with destination Madrid is excluded from all analysis.

^b^The means, medians, standard deviations and percentages in the IPSOS data are calculated using the sample weights.

Numbers of missing values in the two surveys: ^c^5 & 13 (for the latter, age group was recorded but not numeric age); ^d^45 & 0; ^e^100 & 724.

The study compared the CAA survey group to the UK population IPSOS-MORI group who had, in the past, visited a malaria-endemic country and found a larger proportion of the CAA group visiting West Africa (42 *vs* 16%). Other variables, including reason for travel and chemoprophylaxis use, were of similar proportions in the two groups. There was a larger proportion of ethnically black (39 *vs* 4%) travellers interviewed in the CAA survey (Table 2). In the IPSOS survey, the malaria knowledge score in those who had travelled was 63.8 (Table 2), while the scores in those who had not travelled were more than five points lower at 58.6 (95% CI for the difference is 3.9-6.5, p < 0.001).

**Table 2:** Travellers description, itinerary, knowledge prophylaxis use, and source of advice

	**CAA**		**IPSOS**^**a**^	
Region to which travelled: n (%)^b^				
West Africa	208	(42)	95	(16)
South and East Africa	199	(40)	196	(38)
Asia and other	92	(18)	397	(74)
Reason for travel: n (%)^c^				
Business	98	(20)	95	(18)
Leisure	397	(80)	458	(84)
of which^d^: Visiting Friends & Relatives	*128*	*(56)*	*185*	*(27)*
other leisure	*100*	*(44)*	*277*	*(57)*
Prophylaxis: n (%) ^e^				
Yes	283	(61)	302	(57)
No	181	(39)	246	(43)
Knowledge Score (mean & SD) ^f^	70.7	(14)	63.8	(12)
Advice Score: n (%)				
none	121	(24)	113	(18)
non-professional	20	(4)	34	(7)
professional	358	(72)	401	(74)
Ethnicity: n (%) ^g^				
White	75	(51)	393	(82)
Black	57	(39)	33	(4)
Asian	12	(8)	107	(13)
Mixed and other	3	(2)	14	(2)
*Total*	499		548	

^a^Restricted to the 548 reporting previous travel to malaria-endemic countries. The means, standard deviations and percentages are calculated using the sample weights. Percentages may sum to more than 100 due to multiple trips.

^b^5 people in the IPSOS survey did not remember to which malaria-endemic country they had travelled

Numbers of missing values in the two surveys: ^c^ 4 & 3, ^e^35 & 0, ^g^4 & 0, ^g^352 & 1

^d^In the CAA survey, “Migration” (n = 2) and “Studies private/grants -formal academic course” (n = 16) were classified as leisure. This is retained here because follow-on questions were asked on the basis of this classification. For consistency, in IPSOS, ‘live there’ (n = 4) was classified as leisure. There was no education option in IPSOS. In CAA, only 228 of 398 respondents had data on type of leisure

The quality of pre-travel advice in the population who had travelled previously (IPSOS survey) and those who were about to depart (CAA survey) was similar, with over 70% having received professional advice and 18 and 24% not seeking/received pre-travel advice (Table 2). In the subgroup analysis of visitors to Ghana, Nigeria and Kenya, 22, 40 and 23% of travellers, respectively, had not sought or received advice, or used non-professional pre-travel advice for the current trip (Table 3).

**Table 3:** The three most frequently visited countries in the CAA survey

	**Ghana N = 81**	**Difference or regression coefficient** **(95% CI)**	**Nigeria** **N = 86**	**Difference or regression coefficient** **(95% CI)**	**Kenya N = 132**	**Difference (95% CI)**
Reason for Travel, n (%)^a^						
Business	14 (18)		27 (32)		22 (17)	
Leisure	66 (82)		58 (68)		109 (83)	
Of which^b^: VFR	*31 (74)*		*39 (74)*		*23 (34)*	
Other leisure	*11 (26)*		*14 (26)*		*44 (66)*	
Knowledge score (mean) by reason						
Business	74.5		70.8		72.9	
Leisure	70.4	-4.1 (-15, 6.7)	71.7	0.9 (-6.2, 8.0)	69.8	-3.0 (-9.4, 3.4)
Perceived fatality threat of malaria^c^						
Never kills	0 (0)		2 (2)		0 (0)	
Occasionally kills	44 (56)		38 (48)		61 (47)	
Often kills	30 (38)		37 (46)		66 (51)	
Always kills	5 (6)		3 (4)		3 (2)	
Number of trips over the last 12 months on this route^d^						
0	47 (63)		23 (28)		100 (77)	
1 or 2	14 (19)		26 (32)		18 (14)	
≥3	14 (19)		32 (40)		12 (9)	
Chemoprophylaxis regimen, n (%)						
Any prophylaxis^e^	62 (82)		38 (50)		98 (78)	
Of which						
Doxycycline (daily)	14 (18)		4 (5)		19 (15)	
Mefloquine(weekly)	17 (22)		4 (5)		2 (2)	
Atovaquone/proguanil (daily)	28 (37)		24 (32)		72 (57)	
Chloroquine (weekly)	2 (3)		6 (8)		0 (0)	
Other	1 (1)		0 (0)		5 (4)	
Knowledge score by prophylaxis use (mean)						
Prophylaxis not used	77.5		74.2		74.1	
Prophylaxis used	69.8	-7.7 (-18, 2.9)	69.3	-4.9 (-12, 2.0)	69.1	-5.0 (-11, 0.8)
Change in knowledge score per doubling in income		-1.41 (-3.62, 0.79)		0.65 (-1.29, 2.60)		-0.31 (-1.92, 1.3)
Advice obtained by prophylaxis use						
No prophylaxis, n (%)						
None, or non-professional	13 (93)		27 (71)		16 (57)	
Professional	1 (7)		11 (29)		12 (43)	
Prophylaxis, n (%)						
None, or non-professional	4 (6)		5 (13)		10 (10)	
Professional	58 (94)		33 (87)		88 (90)	
All travellers by advice						
None, or non-professional^f^	18 (22)		34 (40)		30 (23)	
Professional	63 (78)		52 (60)^g^		102 (77)^h^	

^a^1 missing value for each country; missing values excluded from denominator of percentages in this & other rows.

^b^N without information on type of leisure, by country: 24, 5 & 42.

^c^N missing by country: 2, 6 & 2.

^d^N missing by country: 6, 5 & 2.

^e^N missing 5, 10 & 6.

^f^These two categories were merged due to small numbers in the latter.

^g^17% less than Ghana, 95% CI 2–32, p = 0.02.

^h^0.5% less than Ghana, 95% CI 12–13, p = 1.

Knowledge in travellers using chemoprophylaxis was very similar to those not using chemoprophylaxis in both the CAA and IPSOS surveys although the IPSOS survey group had a lower average knowledge score, with West Africans having significantly higher scores than other Africans and Asian travellers (Table 4). Comparing the knowledge scores of those who had received professional pre-travel advice with those who were self-informed or those who received no advice, the scores were almost the same. This finding was replicated in the IPSOS survey. Ethnicity provided a mixed picture, with the IPSOS survey respondents having lower knowledge levels than the CAA survey respondents and with Asians in the CAA scoring highest (81) while the mixed and other ethnic groups in the IPSOS survey scored lowest (57). Knowledge scores were not influenced by reason for travel or household income.

**Table 4:** Knowledge score in subgroups of the two surveys

		**CAA**			**IPSOS**			**IPSOS v CAA**
**Variable**		**Mean know-ledge score**	**Difference in means, or regression coefficient**	**95% CI within variable**	**Weighted mean knowledge score**	**Difference in means, or regression coefficient**	**95% CI within variable**	**Overall estimate & CI**^**a**^
Prophylaxis	No	71.7			62.5			
	Yes	70.0	-1.7	(-4.30, 0.98)	64.7	***2.3***	***(0.22, 4.35)***	0.79 (-0.83, 2.41)
Advice score	None	72.8			61.9			
	Non-professional	70.5			62.8			
	Professional	70.0	-2.7	(-5.61, 0.26)	64.3	2.4	(-0.13, 4.97)	0.23 (-1.69, 2.15)
Ethnicity	White	70.2			64.2			
	Black	73.4	3.1	(-1.13, 7.42)	66.1	2.5	(-2.87, 7.76)	2.87 (-0.44, 6.18)
	Asian	80.8	***10.6***	***(3.04, 18.20)***	61.1	-2.6	(-5.57, 0.41)	(-)
Mixed & other		76.7	6.4	(-7.89, 20.80)	56.6	***-7.6***	***(-15.1, -0.05)***	-4.51 (-11.2, 2.13)
Region to which travelled^b^								
West Africa		72.3			68.1			
South and East Africa		70.1	-2.3	(-5.03, 0.52)	62.6	-5.5	***(-10.4, -0.56)***	***-3.04 (-5.45, -0.62)***
Asia and other		68.5	***-3.8***	***(-7.37, -0.34)***	63.3	-4.8	***(-9.34, -0.27)***	***-4.21 (-6.97, -1.44)***
Reason for Travel	Business	72.5			63.5			
	Leisure	70.1	-2.4	(-5.60, 0.73)	63.8	0.3	(-2.77, 2.24)	-0.77 (-2.72, 1.18)
Income (per doubling)			0.34	(-0.41, 1.10)		0.02	(-0.78, 0.81)	0.19 (-0.36, 0.73)

Estimates whose 95% confidence exclude the null value, equivalent to p < 0.05, are shown in ***bold italics.***

^a^Meta-analysis combination of the estimates from the two surveys. This was not done (shown ‘-’) if the two 95% confidence intervals did not overlap.

^b^IAll CAA participants are included. In the IPSOS survey, only those who had travelled to a single region were included here (N = 424).

The use of chemoprophylaxis in passengers travelling to malaria-endemic countries in all or part of their destination was collected. Of the passengers departing to Kenya (132), Nigeria (86) and Ghana (81), (71%, 38% and 74%) respectively, reported taking an effective chemoprophylaxis for their travel (Table 3). Examining the chemoprophylaxis regimens being taken, 57% of travellers to Kenya, 37% to Ghana and 32% of travellers to Nigeria were using atovaquone and proguanil. The mean knowledge score in those using chemoprophylaxis was 69 for all three destinations. This was lower than knowledge scores in those using no chemoprophylaxis.

## Discussion

Understanding the knowledge and risk perception that individuals have of malaria is valuable when trying to understand attitudes and practices in the use of preventative measures. The study surveyed the UK general population to provide a background level of knowledge and attitudes and used the population who had previously travelled to a malaria-endemic region (28%, 548) as a comparator with a similarly sized departing passenger population of 500. The average malaria knowledge score of the general UK population who had not travelled to a malaria-endemic country, at 58.6, was significantly lower than in those who had previously travelled or who were travelling (63.8 and 70.7, respectively).

Knowledge, perception of malaria and its threat, and the use of chemoprophylaxis in departing passengers were assessed to determine possible influences on use of chemoprophylaxis. The main focus was on VFR’s who were travelling to sub-Saharan Africa, as this is the group with the highest morbidity from malaria. The ages in the three groups were similar but black travellers and West Africa as a destination, were more frequent in the CAA group, as this was a destination targeted by the CAA surveyors. The source of pre-travel advice among CAA and IPSOS was similar (72 *vs* 74%) received advice from a professional source). Unexpectedly, the levels of knowledge in passengers using chemoprophylaxis were lower to those not using a drug regimen. Similarly, those travellers who had received professional pre-travel advice did not have a higher knowledge score than travellers who had not received advice or received advice through a non-professional source. The same phenomenon was noted in the non-travelling population, who overall, achieved lower knowledge scores.

Comparing travellers to three destinations in sub-Saharan Africa (Table 3), passengers to Kenya were predominantly (83%) travelling for leisure. The majority of leisure travellers to Ghana and Nigeria were VFRs (74%) while only one third of those travelling to Kenya were VFRs. The mean knowledge score in those using chemoprophylaxis was 69 for the three destinations and higher (77.5, 74.2 and 74.1) in those not using chemoprophylaxis. The perception of threat from malaria was similar in the three groups travelling, with 38% of passengers to Ghana, 46% to Nigeria and 51% to Kenya believing malaria often killed.

When asked about use of chemoprophylaxis, the majority of passengers to Ghana (74%) and Kenya (71%) were taking an effective regimen. However, among passengers to Nigeria, which included 32% travelling on business, only 50% were using any chemoprophylaxis and only 38% used an effective regimen. Despite having similar levels of knowledge and equal understanding of the seriousness of malaria, significantly fewer Nigerians were using preventative drugs for malaria than other destination passengers. There is some evidence that as an alternative to chemoprophylaxis, some VFR travellers choose to self-treat their fever symptoms with anti-malarial drugs either purchased before or during travel [10,11]. This practice of self-treatment is not recommended in the UK national guidelines for travel to high risk regions in sub-Saharan Africa, but may be adopted by semi-immune travellers as an alternative to taking chemoprophylaxis. Nigerians travelled more frequently than passengers to Kenya and Ghana with 72% having made one or more trips to Nigeria in the previous 12 months. Forty percent of Nigerians had not received professional pre-travel advice before the current journey, nearly double that of visitors to Ghana (22%) and Kenya (23%).

The proportion of travellers accessing health care pre-travel in this study represents an unbiased sample of departing travellers and their prophylaxis use. Many other studies of malaria prophylaxis compliance have been based on travellers with malaria or travellers departing from endemic countries [10,12] although some have questioned departure lounge passengers [13].

The study’s assessment of knowledge was based around a range of 22 questions that had weighted responses. It is possible that questions may not have been probing or detailed enough or the weighting not adjusted to provide the sensitivity to define knowledge accurately. The sample sizes of travellers to sub-Saharan regions were large enough for sub-analysis but there were a number of other countries visited in Southeast Asia, the Indian Subcontinent and South America whose numbers were too small to analyse separately.

These findings suggest that malaria knowledge, perceived threat, previous travel experience and source (quality) of pre-travel advice are not important factors in predicting the use of chemoprophylaxis. Failure to obtain pre-travel advice may still be a contributory factor to non-use of chemoprophylaxis but not linked to knowledge of malaria or threat perception, (belief that malaria occasionally, often or always kills) which was similar in the three groups. Repeated travel to Nigeria was strikingly more frequent and may be an important influence in reducing chemoprophylaxis use.

In a European-wide departure survey of travellers to high-risk malaria regions, 56% were carrying malaria prophylaxis and 28% carried self-treatment for malaria [14]. In this survey 52.1% had received pre-travel advice although only 31.4% of VFRs had received advice. The majority of advice (80%) was from a health professional with one-quarter of respondents obtaining advice from the internet and/or friends [14]. A Spanish departure survey reported that 64% of travellers to sub-Saharan Africa were using chemoprophylaxis. Over 83% of respondents had sought pre-travel advice and travellers who had visited the same region previously were three times less likely to seek health information than first-time travellers [15]. Schilthuis interviewed Nigerians and Ghanaians living in the Netherlands and reported that only 16% of the VFRs had adequate knowledge of malaria [5]. Departing Dutch VFR travellers from Schiphol Airport [6] were questioned on pre-travel preparation and use of anti-malaria measures and malaria knowledge; 74% had correct malaria knowledge, a similarly high knowledge score (71) as in this study’s travellers from Heathrow. Dutch passengers’ use of anti-malaria measures was overall lower (31.9%) than that reported by all UK departing passengers (61%) and matched the proportion of Nigerian travellers ‘use of an effective chemoprophylaxis (38%), although the assessment of prophylaxis usage was defined differently across the two studies, and findings may not be directly comparable. These two studies highlight that malaria knowledge and risk awareness combined with receipt of pre-travel advice does not necessarily result in compliance with malaria preventative measures.

Neave and colleagues [10] highlighted this paradox, where for many VFRs the actual malaria risk did not correlate to personal perceived risk of developing malaria, and suggested that chemoprophylaxis use is influenced by perceived susceptibility, previous experience of malaria, cost of chemoprophylaxis, threat of fatality from malaria and peer pressure [16]. This study finds that perception of fatality from malaria does not correlate with uptake, as Nigerians and Ghanaians have the same perceived threat but very different chemoprophylaxis use. Cost of chemoprophylaxis has been argued as a major barrier to prophylaxis uptake, but recent evidence from two London communities is that, despite availability of free chemoprophylaxis for travellers, there was marginal reduction in imported malaria in VFR travellers when compared to a non-subsidized community [17].

## Conclusion

Reducing malaria in high-risk VFR travellers will require other strategies than relying on improving malaria knowledge and enhancing malaria risk awareness. Future strategies that may improve malaria protection might include rapid access to diagnosis with rapid diagnostic tests and artemisinin combination treatment in London primary care practices which would be more rapidly accessible than tertiary care diagnostics and in-patient treatment. A more radical solution could be to selectively provide diagnostic and self-treatment kits to semi-immune adults who should be capable of undertaking their own testing. Such a policy would however be difficult to implement in parallel with the current policy, which promotes the use of chemoprophylaxis.

## Competing interests

Ron Behrens is on the advisory board of Sigma Tau.

## Authors’ contributions

The study was conceived and designed by RHB. Statistical analysis was undertaken by NA. The interpretation of the data was undertaken by both authors, read and approved the final manuscript as was the preparation and final review of the manuscript.

## References

[B1] WHO World Malaria Report 2012 World Health Organization http://www.who.int/malaria/publications/world_malaria_report_2012/en/

[B2] Smith AD Bradley DJ Smith V Blaze M Behrens RH Chiodini PL Whitty CJ Imported malaria and high risk groups: observational study using UK surveillance data 1987–2006 BMJ 2008 12 103 106 10.1136/bmj.a120 PMC2453297 18599471

[B3] Pistone T Guibert P Gay F Malvy D Ezzedine K Receveur MC Siriwardana M Larouze B Bouchaud O Malaria risk perception, knowledge and prophylaxis practices among travellers of African ethnicity living in Paris and visiting their country of origin in sub-Saharan Africa Trans R Soc Trop Med Hyg 2007 12 990 995 10.1016/j.trstmh.2007.05.009 17643457

[B4] Leonard L VanLandingham M Adherence to travel health guidelines: the experience of Nigerian immigrants in Houston, Texas J Immigr Health 2001 12 31 45 10.1023/A:1026610602073 16228800

[B5] Schilthuis HJ Goossens I Ligthelm RJ de Vlas SJ Varkevisser C Richardus JH Factors determining use of pre-travel preventive health services by West African immigrants in The Netherlands Trop Med Int Health 2007 12 990 998 10.1111/j.1365-3156.2007.01856.x 17697094

[B6] van Genderen PJ Van Thiel PP Mulder PG Overbosch D Trends in the knowledge, attitudes and practices of travel risk groups towards prevention of malaria: results from the Dutch Schiphol Airport Survey 2002 to 2009 Malar J 2012 12 179 10.1186/1475-2875-11-179 22642661 PMC3413569

[B7] Chiodini PL Field VK Hill DR Whitty CJM Lalloo DG Guidelines for Malaria Prevention in Travellers from the United Kingdom. London, Public Health England, July 2013 http://www.hpa.org.uk/web/HPAweb&HPAwebStandard/HPAweb_C/1203496943315

[B8] 2009 CAA Passenger Survey Heathrow Airport http://www.caa.co.uk/docs/81/LHR2009.pdf

[B9] Cleophas TJ Zwinderman AH Meta-analysis Circulation 2007 12 2870 2875 10.1161/CIRCULATIONAHA.105.594960 17548743

[B10] Neave PE Jones CO Behrens RH A review of risk factors for imported malaria in the European African diaspora J Travel Med 2010 12 346 350 10.1111/j.1708-8305.2010.00440.x 20920057

[B11] Wieten RW Harting J Biemond PM Grobusch MP Van Vugt M Towards improved uptake of malaria chemoprophylaxis among West African travellers: identification of behavioural determinants Malar J 2013 12 360 10.1186/1475-2875-12-360 24107150 PMC3852732

[B12] Ropers G Holle DRB Wichmann O Kappelmayer L Stuben U Schonfeld C Stark K Determinants of malaria prophylaxis among German travelers to Kenya, Senegal, and Thailand J Travel Med 2008 12 162 171 10.1111/j.1708-8305.2008.00188.x 18494693

[B13] Dahlgren AL DeRoo L Steffen R Prevention of travel-related infectious diseases: knowledge, practices and attitudes of Swedish travellers Scand J Infect Dis 2006 12 1074 1080 10.1080/00365540600868354 17148080

[B14] Van Herck K Zuckerman J Castelli F Van Damme P Walker E Steffen R European Travel Health Advisory B Travelers' knowledge, attitudes, and practices on prevention of infectious diseases: results from a pilot study J Travel Med 2003 12 75 78 12650648 10.2310/7060.2003.31638

[B15] Lopez-Velez R Bayas JM Spanish travelers to high-risk areas in the tropics: airport survey of travel health knowledge, attitudes, and practices in vaccination and malaria prevention J Travel Med 2007 12 297 305 10.1111/j.1708-8305.2007.00142.x 17883460

[B16] Neave PE The burden of imported malaria among Nigerians and Ghanaians living in London: Understanding the influences of the social, cultural, environmental, economic and structural context PhD. Thesis 2013 London: London School of Hygiene and Tropical Medicine, Dept of Infectious and Tropical Diseases

[B17] Neave PE Taylor S Behrens RH Does public subsidy of the cost of malaria chemoprophylaxis reduce imported malaria?A comparative policy analysis Malar J 2013 12 238 10.1186/1475-2875-12-238 23848986 PMC3723845

